# Are Participants in a Street-Based HIV Testing Program Able to Perform Their Own Rapid Test and Interpret the Results?

**DOI:** 10.1371/journal.pone.0046555

**Published:** 2012-10-08

**Authors:** Luis de la Fuente, María Elena Rosales-Statkus, Juan Hoyos, José Pulido, Sara Santos, María José Bravo, Gregorio Barrio, Sonia Fernández-Balbuena, María José Belza, the Madrid Rapid HIV Testing Group

**Affiliations:** 1 Centro Nacional de Epidemiología, Instituto de Salud Carlos III, Madrid, Spain; 2 CIBER Epidemiología y Salud Pública, Madrid, Spain; 3 Servicio de Medicina Preventiva, Hospital Central de la Cruz Roja San José y Santa Adela, Madrid, Spain; 4 Escuela Nacional de Sanidad, Instituto de Salud Carlos III, Madrid, Spain; Rollins School of Public Health, Emory University, United States of America

## Abstract

**Objective:**

Availability of over-the-counter rapid HIV tests could improve access to testing those reluctant or unable to use current services. We aimed to evaluate the feasibility of HIV self-testing using a finger-stick whole-blood rapid test (Determine™ HIV Combo) to detect both antigen and antibody.

**Methods:**

Before being tested, 313 participants in a street-based testing program were given adapted instructions and a test kit, and performed the self-test without supervision. These participants, together with another 207 who performed supervised self-testing, received additional instructions on how to interpret the test results shown in six colour photos and filled out a questionnaire. Logistic regression and generalized estimating equations (GEE) were used in the statistical analysis.

**Results:**

About 8.0% (95%CI:4.8%–11.2%) obtained an invalid self-test. An invalid result was inversely associated with male participants who had sex with men (OR = 0.3;95%CI:0.1–1.0). Of the 3111 photos interpreted,4.9% (95%CI:4.1–5.7) were incorrect. Only 1.1% (95%CI:0.3–1.8) of the positive results were interpreted as negative. Age 30 or older (OR = 2.1; 95%CI:1.2–3.7), having been born in Latin America (OR = 1.6; 95%CI:1.1–2.2),and not having university education (OR = 2.1;95%CI:1.2–3.7) were associated with misinterpreting test results in the GEE. Participant's perceptions of both their proficiency when conducting the test and interpretation were related with actual outcomes. Most participants (83.9%) were more motivated than before to use the self-test in the future, and 51.7% would pay >10 Euros for the test if it was sold in pharmacies.

**Conclusions:**

This is the first study showing that blood-based self-testing with current technology is feasible in HIV-negative participants demanding the test and without prior training or supervision. Bearing in mind that it was conducted under difficult weather conditions and using a complex kit, over-the-counter tests could be a feasible option to complement current diagnostic strategies. More studies are needed to accommodate technology, minimise interpretation mistakes and provide on-line support.

## Introduction

It is estimated that one-third of HIV-infected persons in the European Union [1] and one of every five in the United States do not know their serological status [2]. Those diagnosed with advanced immune deficiency are known to suffer greater morbidity and mortality [3], [4]. Moreover, persons who are unaware of their infection have a transmission rate 3.5 times higher than those who know their serological status [5]. Thus, the earliest possible diagnosis is one of the most efficient strategies to control the epidemic, and is currently a priority of all infection control plans.

Testing policies are undergoing extensive review in many countries [6]–[8] and international agencies [9], [10]. All new versions are moving toward ending the “exceptionalism” that previously guided them. Among the new strategies is the use of “rapid” and “point of care tests” (POC) in different contexts, including non-clinical settings. These tests are easy to use and provide results within 1 to 40 minutes. The ones available require samples of oral fluid or blood. The oral fluid-based tests are less sensitive than the blood-based ones [11]. Moreover, some oral fluid-based programs have resulted in an unacceptable percentage of false positives [12], [13]. Although lower than in current reference laboratory EIAs, specificities and sensitivities of HIV rapid test kits are high [14] and are comparable to first and second generation conventional EIAs. However, reduced sensitivity in early stages of infection could lead to false negative results [15]. The 4^th^ generation immunoassay (IA), also incorporates detection of p24 antigen. P24 detection is useful to shorten the window period but makes it more difficult to interpret the result. This test is used by several NGOs running diagnostic programs in outreach and community settings in Spain, and has been chosen for this study in view of the concerns raised by the results obtained with oral fluid tests and the possibility of obtaining an earlier HIV diagnosis with the detection of the HIV-1 antigen.

Rapid tests could be sold over the counter for self-testing, much like pregnancy tests. Some authors have thought that self-testing can be an innovative component of community-wide HIV-prevention strategies [16], [17] by providing testing to persons who for reasons of stigma or confidentiality do not wish to reveal their sexual practices; such persons could be tested when they perceive they are at risk of infection – something they may not feel they need again for a long time. Some authors have commented that if diagnostic technology is adequate, it is difficult to justify restricting access to it [18]. Others believe that authorisation of over-the-counter sale of these tests may cause more problems than they could solve [19]. What is clear is that there are no published studies that analyse to what extent individuals are able to carry out self-testing without training or assistance and under the stress caused by the immediacy of a possible positive result. Only four studies were conducted addressing this topic, but one did not incorporate interpretation and included only seropositive individuals [20], and another was conducted mostly in this group and showed disappointing results [21]. The third and fourth study convey very good results, but participants either had seen how they were tested immediately before they performed their own self-test [22], or performed their own test after a demonstration done by a counsellor [23]. Currently there are no licensed rapid home tests for HIV in European countries but the U.S. Food and Drug Administration (FDA) has just approved an over the counter rapid HIV test using oral fluid, based on trials conducted by the manufacturer [24].

The present study evaluated the feasibility of HIV self-testing using a finger-stick whole-blood combo rapid test – including obtaining the sample and interpreting the results in conditions similar to those that would be found if this technology were available over the counter.

## Methods

The study was nested in a public health program operated by the non-governmental organisation “Madrid Positivo”. This program has been running since 2008 with the objective of promoting HIV early diagnosis. It is carried out, at irregular intervals during the year, in a mobile unit operating mainly in the Region of Madrid but also reaching out to other Spanish cities. The rapid test used in the program was initially a blood-based test, Determine HIV 1/2 test, which was replaced in 2009 by Determine HIV-1/2 Ag/Ab Combo.

A cross-sectional study was conducted between October 2009 and February 2010 (a particularly cold and rainy winter), in tents located outdoors. Participants were recruited from persons requesting a rapid HIV test in five different places of three cities in the Region of Madrid: a street near the “gay neighbourhood”, two university campuses, and two squares near railway stations.

The blood-based test Determine HIV-1/2 Ag/Ab Combo was used in the program and was also used in the study (hereafter, program and study test). This test detects both p24 antigen and anti-HIV antibodies. Testing in the program was carried out following the manufacturer's instructions, but two modifications were introduced for the study. First, we did not provide the capillary tube to participants, since the experience of the study of Lee et al [21], and our qualitative pilot study showed that a large percentage of persons were unable to use it correctly. It was decided instead to ask participants to deposit “at least two large drops” of blood directly in the appropriate place on the reactive strip. This amount would guarantee the volume of 50 µl indicated by the manufacturer and considered to be equivalent to a drop of blood and would also take into account variations existing among different subjects. We were not absolutely certain that this method would work, but our observations during the pilot study showed good results. The second modification was to mount the strip on a support indicating the place where the blood should be deposited. Ad-hoc instructions on how to carry out the whole process were also developed, after repeated simulation of the process with volunteers using a non-reactive test. Two explanatory brochures were developed: one on how to perform the self-test and the other on how to interpret the results.

**Figure 1:**
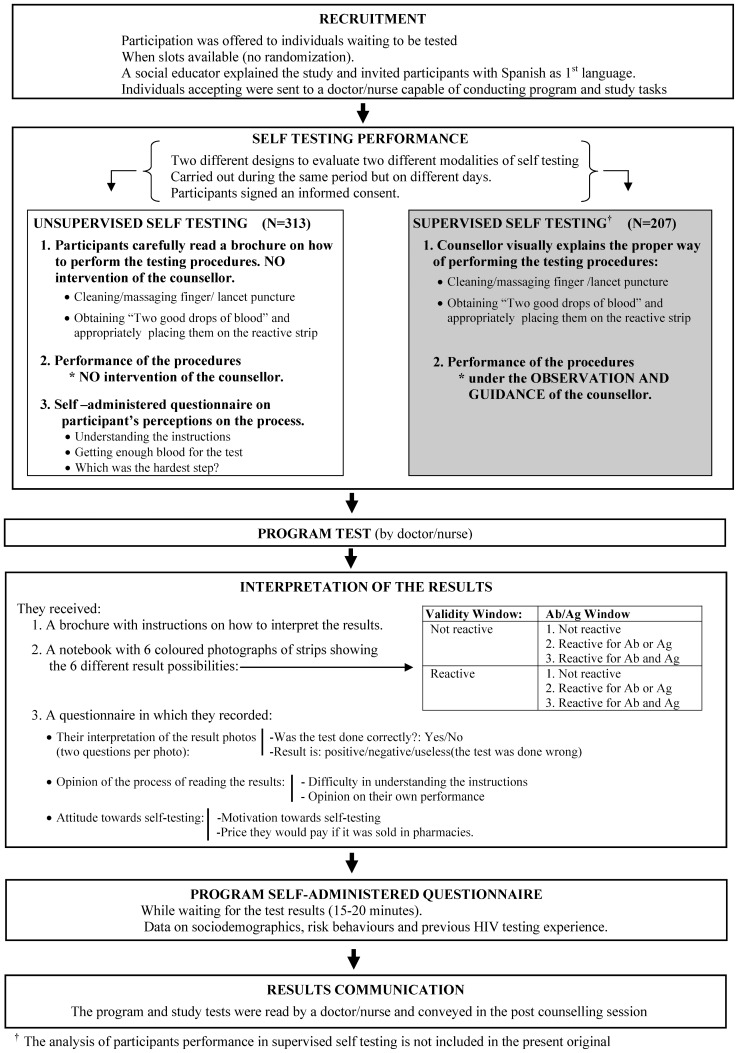
Sequence of processes followed by the participants.

While awaiting the program test, a social educator explained the study and invited clients who had Spanish as their first language to participate. They were not offered any monetary incentive. Those who accepted and said they had not participated in the study before were sent to a doctor or nurse who could conduct both the counselling tasks for the study and all tasks for the program, in accordance with the requirements of Spanish legislation. The doctor/nurse again explained the study objectives and procedures, and participants signed an informed consent form. The study protocol was approved by the institutional review board of the Instituto de Salud Carlos III without any request for prior approval from the Spanish Agency of Medicines and Medical Devices (AEMPS). Because the study test had to be performed before the program test to avoid any learning process, the sequence of procedures was as follows: (see figure 1).

1. *Performance of the self-test:* 313 participants were given a brochure with written instructions supported by illustrations, and were instructed to read it carefully before carrying out the procedures (cleaning and massaging the finger, lancet puncture, obtaining “two large drops of blood” ,and depositing them in the appropriate place on the reactive strip). They were then given a kit with the materials to perform the test, as if they had bought it in a pharmacy and were doing it at home, with no explanation from the doctor or nurse, who only observed. Another 207 participants performed self-testing with the same materials, but after brief training and under the supervision of a counsellor. They participated in the procedure for interpretation of results described below, however, this methodology had a different objective, and the results with valid-invalid tests obtained with this supervised procedure have been presented elsewhere [25] and are not reported in the present study. Both designs were carried out during the same period but on different days. Despite weather conditions, the temperature inside the tents was maintained within the temperature ranges prescribed by the test's manufacturer.
2. Opinion of the *self-testing process:* the self-administered paper questionnaire asked about the difficulty in understanding the instructions, if they thought they had deposited “two large drops of blood in the correct place” and which part of the process had been most difficult.
3. *Performance of the program test:* The doctor/nurse counsellor performed the test under the same conditions for every client, following the manufacturer's instructions, including the use of a capillary tube. The reactive strip of both the study and the program tests were placed out of sight of the participants, to prevent any interference with the remaining procedures and to be able to present the results at the end of the entire process.
4. *Evaluation of the interpretation of results:* Neither the persons who performed the self test without supervision nor those who participated in the other methodology not included in the present study, interpreted the result of their own self-test. Rather, they each received a brochure with instructions, a booklet showing six photographs of reactive strips of other tests, and a questionnaire to record the result. Each photo showed a different situation: three were invalid and three were valid, and in each case the photos included one with no reactivity in either the Ag or Ab window; one with reactivity in the Ag or in the Ab window, and another reactive in both. To evaluate their interpretation of the result two questions were asked: a) Was the test done correctly? (yes, it was done well; no, it was wrong); b) then the result is (positive/negative/useless, because the test was done wrong. The participants' individual interpretation of six different test photographs permitted collection of sufficient data to analyse the correct interpretation of the more infrequent results, especially those that were positive.
5. Opinion of the *process of reading the results*, based on two questions: difficulty in understanding the instructions and opinion as to whether the participant had performed the interpretation of results correctly.
6. *Attitude about the self-test:* whether, after experiencing the process of self-testing, the participant felt more or less motivated to use it in the future, and the price that he/she would be willing to pay if it were sold in pharmacies.
7. *Program self-administered questionnaire:* This was completed by all participants, whether or not they were involved in the study, during the waiting time required to read the test result (20–30 minutes). The questionnaire was intended to collect demographic and risk behaviour data, as well as previous HIV testing experience. The test results, for both the program and the study, were read by the doctor/nurse and conveyed in the post-counselling session.

Several mechanisms and program conditions made it unlikely that a subject could participate in the study more than once: the social educator asked about previous participation, the doctor/nurse had the capability to identify those who had not been filtered before, and participants had to sign an informed consent form; furthermore, monetary incentives were not offered, and the time spent in the study lengthened the entire HIV diagnosis process.

### Statistical Analysis

All the analyses were exploratory and descriptive, without a priori quantified hypotheses. To evaluate the proportion of subjects with an invalid test and to determine their correlates, we analysed the 313 persons (83.0%) who performed the self-test of the 377 who were invited to participate. To analyse the factors associated with obtaining an invalid test, we calculated the odds ratio (OR), its 95% confidence interval (CI), and the statistical significance with the Chi-square, and we planned to developed a logistic regression model that included those variables with p<0.1.

For the interpretation of results we considered the 519 participants who carried out this process, 81.0% of the 641 who were invited to participate. This includes 207 participants who performed the supervised self-test. Each participant interpreted not just one photo, but six. For this reason, the underlying correlated nature of the data was taken into account when calculating the CIs for the percentage of results photos that were misinterpreted as well as the unadjusted and adjusted ORs [26]. Unadjusted and adjusted odd ratios were calculated by a repeated-measures logistic model with population-average estimates of the effects (ORs) and robust standard errors [27]. To evaluate the interpretation that the participants would have made of the results of their own self-test, we considered their interpretation of the photograph in the booklet equivalent to the result obtained in their own self-test, as the latter was not interpreted by the participant but only by the doctor/nurse. It was assumed that participants with an invalid result and correct interpretation of the corresponding picture would have had to repeat the rapid test. To evaluate how well they would perform on this second test, we analysed their performance in interpreting the picture that corresponded to the results obtained in the program test.

## Results

Sociodemographic data, risk behaviours and history of previous HIV testing are shown in table 1. There were no significant differences between those who accepted and those who refused participation in the study.

**Table 1:** Demographic, behavioural characteristics and HIV testing experience of participants and of those who refused to participate in the Madrid HIV self-testing study.

	Self-testing performance					Interpretation of test results				
	Participants		Refused to participate			Participants		Refused to participate		
	(N = 313)		(N = 64)			(N = 519)		(N = 122)		
	N	%	N	%	*p*-value	N	%	N	%	*p*-value
**Place of testing**					0.196					0.074
Campuses in Madrid	59	18.8	13	20.3		133	25.8	38	31.1	
A square in the gay quarter in Madrid	161	51.4	39	60.9		290	56.2	72	59.0	
Other cities of the Region of Madrid	93	29.7	12	18.8		93	18.0	12	9.8	
**Age group**					0.112					0.251
<30	172	56.2	41	67.2		301	59.8	78	65.5	
≥30	134	43.8	20	32.8		202	40.2	41	34.5	
**Gender/Sexual behaviour**					0.093					0.526
Women	91	29.5	27	43.5		175	34.2	47	39.2	
Heterosexual men	106	34.4	16	25.8		149	29.1	30	25.0	
Men who have sex with men	111	36.0	19	30.6		188	36.7	43	35.8	
**Country of birth**					0.523					0.597
Spain	260	83.9	54	87.1		432	83.9	103	85.8	
Latin America	50	16.1	8	12.9		83	16.1	17	14.2	
**Level of education**					0.924					0.372
University	159	51.5	32	50.8		278	54.1	60	49.6	
<University	150	48.5	31	49.2		236	45.9	61	50.4	
**Country of residence (last 12 months)**					0.314					0.154
Spain	293	95.8	62	98.4		478	94.3	116	97.5	
Another country	13	4.2	1	1.6		29	5.7	3	2.5	
**Source of income**					0.184					0.401
Employment with/without a contract	257	83.4	55	90.2		413	81.3	99	84.6	
Other	51	16.6	6	9.8		95	18.7	18	15.4	
**Ever injected drugs**					0.536					0.074
No	298	99.3	57	100.0		488	99.0	115	100.0	
Yes	2	0.7	0	0.0		5	1.0	0	.0	
**Previous HIV test**					0.172					0.355
Yes	160	53.0	26	43.3		243	49.0	52	44.4	
No	142	47.0	34	56.7		253	51.0	65	55.6	
**Last HIV test performed**					0.708					0.669
Rapid test	42	13.9	7	12.1		68	13.7	14	12.2	
Conventional test or no previous test	260	86.1	51	87.9		429	86.3	101	87.8	

### Self-testing performance

Of the 313 participants, 25 (8.0%; 95% CI:4.8%–11.2%) obtained a test interpreted by the doctor/nurse as invalid, whereas there were no invalid program tests. In the unadjusted analysis, only two variables were associated with a lower risk of invalid results: having performed the test in a square of the gay quarter in Madrid (OR = 0.3; 95% CI: 0.1–1.0) and being a MSM (OR = 0.3; 95% CI: 0.1–1.0) (table 2). Considering their collinearity (most participants in the gay quarter were MSM), the logistic analysis was unnecessary since place of testing would not be included in the model.

**Table 2:** Factors associated with an invalid result on performance of the self-test and misinterpretation of a test result.

	Self-testing performance (N = 313)					Interpretation of test results (N = 519)									
	Unadjusted analysis					Unadjusted analysis					Adjusted analysis				
	OR	95% CI			p-value	OR	95% CI			p-value	aOR^a^	95% CI			p-value
**Place of testing**															
Campuses in Madrid	1.0					1.0									
A square in the gay quarter in Madrid	0.3	0.1	-	1.0	0.044	1.8	0.8	-	4.1						
Other cities of the Region of Madrid	1.6	0.6	-	4.3	0.388	1.5	0.6	-	3.6						
**Age group**															
<30	1.0					1.0					1.0				
≥30	1.0	0.4	-	2.3	0.982	2.0	1.2	-	3.3	0.013	2.1	1.2	-	3.7	0.008
**Gender/Sexual behaviour**															
Women	1.0					1.0									
Heterosexual men	1.3	0.5	-	3.1	0.599	1.7	0.8	-	3.3	0.149					
Men who have sex with men	0.3	0.1	-	1.0	0.044	1.7	0.8	-	3.3	0.139					
**Place of birth**															
Spain	1.0					1.0					1.0				
Latin America	1.7	0.7	-	4.6	0.269	2.5	1.3	-	4.6	0.004	1.6	1.1	-	2.2	0.006
**Level of education**															
University	1.0					1.0					1.0				
<University	1.4	0.6	-	3.2	0.438	1.7	1.0	-	3.0	0.048	2.1	1.2	-	3.7	0.013
**Place of residence (last 12 months)**															
Spain	1.0					1.0									
Another country	0.9	0.1	-	7.5	0.949	0.3	0.1	-	1.0	0.049					
**Source of income**															
Employment with/without a contract	1.0					1.0									
Other	0.4	0.1	-	1.9	0.272	0.6	0.3	-	1.3	0.224					
**Ever injected drugs**															
No	b					1.0									
Yes						5.4	1.2	-	23.9	0.027					
**Previous HIV test**															
Yes	1.0					1.0									
No	1.0	0.4	-	2.2	0.903	0.9	0.5	-	1.5	0.619					
**Last HIV test performed**															
Rapid test	1.0					1.0									
Conventional test or no previous test	1.8	0.4	-	8.2	0.418	1.1	0.4	-	2.9	0.809					
**Type of self-test**															
Unsupervised self-testing						1.0									
Supervised by a counsellor						1.0	0.6	-	1.8	0.906					

a aOR = adjusted odd ratio.

b Not calculated due to low prevalence of injection.

### Interpretation of test results

**Figure 2:**
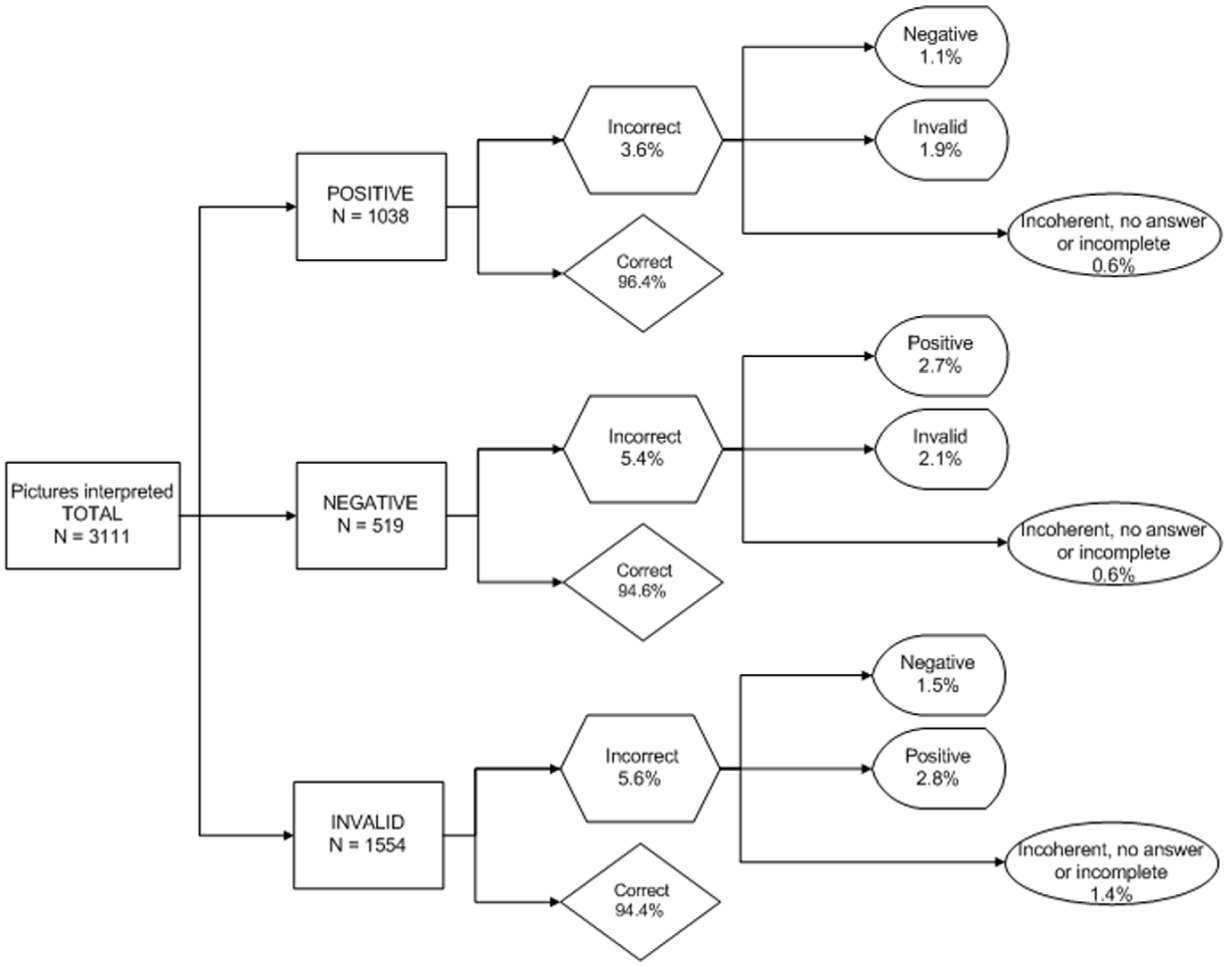
Errors committed in the interpretation of results photos according to the three possible situations.

The 519 participants interpreted a total of 3111 photos, 152 of which (4.9%;95% CI: 4.1–5.7) were interpreted incorrectly. About 14.3% of the participants (95% CI: 11.2–17.4) misinterpreted at least one. Five people misinterpreted four, one person misinterpreted five, and three misinterpreted all (including one person who was overwhelmed with fear of a positive result and was unable to attempt the interpretation). One person could not participate in the interpretation of results because he became dizzy while performing the test. The photos with a positive result had the fewest mistakes (3.6%, 95% CI: 2.2–4.9), although this was not significantly different from those with negative (5.4%, 95% CI: 3.4–7.3) or invalid results (5.6%, 95% CI: 4.2–7.4). The results with the most serious consequences, evaluating a positive test as negative or an invalid test as negative, occurred for 1.1% (95% CI: 0.3–1.8) and 1.5% of photos (95% CI: 0.8–2.3), respectively (figure 2).

In the GEE multivariate analysis, the factors that remained independently and significantly associated with misinterpreting test results were age 30 or older (OR = 2.1; 95% CI: 1.2–3.7), having been born in Latin America (OR = 1.6; 95% CI: 1.1–2.2), and not having university education (OR = 2.1; 95% CI: 1.2–3.7) (table 2).

About 5.6% (95% CI: 3.4–7.7) of participants incorrectly interpreted the result of the photo corresponding to the result of their own self-test, including one HIV-positive person who thought the test was done wrong, although in the second question this person stated that it was positive (table 3).

**Table 3:** Interpretation of the picture equivalent to the participant's own test result.

	Self-test result						Self-test or programme result^a^					
	Participants	Picture wrongly interpreted					Participants	Picture wrongly interpreted				
	N	N	%	(95% CI)			N	N	%	(95% CI)		
Negative result	485	27	5.6	(3,4	-	7,7)	510	28	5.5	3.4	-	7.6
Positive result	8	1^b^	12.5	(0,3	-	52,7)	9	1^b^	11.1	0.3	-	48.3
Invalid result	26	0	0.0									
TOTAL	519	28	5.4	(3,4	-	7,4)	519	29	5.6	3.5	-	7.7

a We assumed that those participants with an invalid result and correct interpretation of the corresponding picture would have had to repeat the rapid-test. To evaluate how well they would perform on this second test we analysed their performance in interp.

b This person's response was contradictory: he said in the first question that the test was invalid, but in the second question that it was positive.

### Perception and opinions

Significant differences were found in the results of user proficiency when conducting the test and test interpretation depending on how participants perceived these processes (table 4). Thus, 6.6% of those who obtained a valid result believed that the instructions were “somewhat” or “quite difficult”, versus 20% of those with invalid results. Likewise, the proportion of those who thought their performance in obtaining enough blood for the test was “fair”, “poor” or “very poor” was 16% vs. 58.3%, respectively. Opinions about the most difficult step were also different: a larger percentage of those with an invalid result chose obtaining the two drops of blood and depositing them in the correct place (processes highly related with the final result) as the hardest steps. Only 2.1% in the group who interpreted all the results correctly thought that the instructions on how to read the results were “somewhat” or “quite difficult” versus 23% of those who made one or more mistakes, and only 2.3% of the former group stated their performance in reading the results was “fair”, “poor” or “very poor” vs 28.4% of those in the latter group.

**Table 4:** Perception and opinions of participants about the process of self-testing.

	Self-testing performance				Interpretation of test results			
	Valid result N = 288	Invalid result N = 25	Total N = 313		All correct N = 445	≥one mistake N = 74	Total N = 519	
	%	%	%	p-value	%	%	%	p-value
**How did you find the test instructions?**				0.017				
Very easy	45.6	24.0	43.9					
Quite easy	47.7	56.0	48.4					
Somewhat difficult	6.6	20.0	7.7					
Quite difficult	0.0	0.0	0.0					
**How well did you do on getting enough blood for the test?**				0.000				
Very well	38.7	8.3	36.3					
Well	45.3	33.3	44.4					
Fair	12.2	20.8	12.9					
Bad	3.1	16.7	4.2					
Very bad	0.7	20.8	2.3					
**Which one was the hardest step?**				0.076				
Prick myself with the lancet	24.2	4.2	22.7					
Get two large drops of blood	34.7	45.8	35.6					
Place the blood on the right spot	24.2	41.7	25.6					
Other	5.6	4.2	5.5					
None	11.2	4.2	10.7					
**How did you find the reading results instructions?**								0.000
Very easy					66.5	33.8	61.8	
Quite easy					31.4	43.2	33.1	
Somewhat difficult					1.8	20.3	4.5	
Quite difficult					0.2	1.4	0.4	
Very difficult					0.0	1.4	0.2	
**How well do you think you did reading the results?**								0.000
Very well					64.7	23.0	58.6	
Well					33.0	48.6	35.3	
Fair					2.3	24.3	5.5	
Bad					0.0	2.7	0.4	
Very bad					0.0	1.4	0.2	
**After today's experience, what is your opinion on being willing to use the test?**				0.271				0.574
More motivated than before	81.0	95.7	82.1		83.7	85.1	83.9	
Same opinion on being willing to use it	18.3	4.3	17.2		15.6	13.5	15.3	
Same opinion of not wanting to use it	0.7	0.0	0.7		0.7	1.4	0.8	
Less motivated tu use it than before	0.0	0.0	0.0		0.0	0.0	0.0	
**What is the maximum price you would pay for this test?**				0.342				0.004
Zero euros	2.9	4.5	3.1		4.9	7.2	5.2	
1 to 9 euros	19.5	4.5	18.4		20.3	21.7	20.5	
10 to 19 euros	32.4	36.4	32.7		35.5	26.1	34.2	
20 to 29 euros	25.4	27.3	25.5		22.9	17.4	22.1	
30 to 39 euros	10.7	9.1	10.5		10.7	8.7	10.5	
≥40 euros	9.2	18.2	9.9		5.6	18.8	7.4	

Some 83.9% of participants indicated that their opinion after participating in the study had changed and they felt more motivated than before to use the self-test in the future, with no significant differences by test result or interpretation. The maximum price people were willing to pay for this test if it was sold in pharmacies ranged between 0 and 100 euros. About 5.2% of participants were not willing to pay anything, 22.1% were willing to pay 20–29€, and 17.9% would pay 30€ or more.

## Discussion

This is the first published study showing that a high percentage of HIV negative people who go for an HIV test are able to perform a blood-based POC and read the results correctly while waiting to know if they are HIV positive. However, 8% did not achieve a valid test, and about 5% of test results were interpreted incorrectly, both overall (for all six photographs) and for the single photo corresponding to each participant's own test result in the program. 1.1% of positive results photos were interpreted as negative, the type of error with the worst consequences. Misinterpreting test results was higher among people older than 30, Latin Americans, and those without university education. Self-evaluation of their own behaviour in the level of proficiency when conducting the test and interpreting the results was clearly related to the ability to obtain valid tests and to interpret them correctly.

To our knowledge, none of the other published studies on the feasibility of self testing [20]–[23] was carried out without training or assistance and under the stress provoked by the immediacy of a result, included both components of self-testing: performance and interpretation. One [20] was carried out in seropositive persons, another was conducted mostly in this group [21], and, strictly speaking, the last two studies were not self-testing because participants either received a tutorial session given by a counsellor [23] or had already seen how they were tested immediately before they performed their own self-test [22]. Three of these studies [20], [22], [23] employed OraQuick® with all or most of the participants using saliva samples. Only the study by Lee [21] was totally performed with Determine and blood sample.

In our study only 8% did not achieve a valid test, as compared to 56% in the study of Lee [21]. Since there were no invalid results among the program tests, we assume that those obtained in the study test were due to the participants' proficiency in conducting the test. This notable difference was due in large part to our decision to eliminate the capillary tube, since the study of Lee and our pilot studies showed that untrained people find it very difficult to use this device. We instructed participants to obtain and deposit two “large drops” of blood, as an approximation for the 50 µl indicated in the kit instructions. This may seem a crude method and would need to be approved as part of the adaptations to transform the test into a home test kit, but the subsequent validity control shows whether enough blood was deposited in the correct place. Our test performance results cannot be compared with those of Spielberg, Gaydos or Choko [20], [22], [23], given the previously mentioned characteristics of these studies. The fact that MSM had a lower probability of invalid tests was not due to their greater previous experience with similar rapid tests, as we controlled for this variable.

It is difficult to compare our 5% of misinterpreted results with what was found in the aforementioned studies, for various reasons (number of results evaluated per participant, percentage of each possible result -positive, negative, invalid- etc.). We found 1.1% of misinterpretation of the most relevant result (actually positive interpreted as negative), and Lee found 2% [21]. Gaydos [22] and Choko [23] have recently reported almost no mistakes, but in fact Gaydos considered only HIV negative patients and in the case of Choko the study involved a previous brief demonstration. The fact that less educated and older people made more errors in interpretation was to be expected. If, as occurred with home kits in the United States [28], those with a high educational level are the first to find out about this alternative and the most likely to use it, fewer errors of interpretation would occur in practice.

We believe that our results overestimate the percentage of invalid and misinterpreted results that would be obtained under conditions of a home setting since the study was conducted in tents during an especially cold and rainy winter; such conditions might have made it harder for some people to concentrate and take the time needed to understand the instructions and carry out the actual testing process (which includes both, performance and interpretation). Moreover, participants were not paid, and they knew they would be tested again in the program.

It is notable that after having experienced the self-test, 4 out of 5 persons were more motivated to do the test, and no one was less motivated. We do not know the cost of transforming the test into a home kit. In a country that offers free HIV testing, 17.9% of participants said they would be willing to pay 30€ or more for a home self-testing kit, and 22.1% would be willing to pay 20–29€. Omitting the use of the capillary tube by not providing it in the kit drastically reduced the percentage of invalid results in this self-testing study. However, another procedure should be developed that allows people with no type of previous training to be sure they have extracted and used a sufficient amount of blood. We also learned that interpretation of results is complex, since it includes separate evaluation of reactivity to antibodies and to p24 antigen. A positive result could be shown in three different ways: positive antibodies, positive antigen, or positive in both. Moreover, each could occur with or without a validity line. Given this complexity, the small diagnostic gain achieved with the detection of antigen (5–7 days) [15], and the fact that several studies have questioned its sensitivity [11], [29], [30], it is questionable whether the p24 antigen should be included in a self test. The current packaging, labelling and instructions were intended to be used by professionals. Therefore, new packaging and ad-hoc instructions for carrying out the test and interpreting the results should be developed, similar to what was done in this study.

It is difficult to assess the extent to which our study population represents potential users but, unlike other studies, all the participants came to be tested. The participation rate was very high, and there were no significant differences in sociodemographics or risk behaviours between participants and non-participants. Based on procedures and in the absence of association for the variable “type of self test”, we believe the fact that some who participated in interpreting the results had performed supervised self-testing did not affect the results.

Despite the evidence presented in this study on the feasibility of the self-test, further studies are needed in other populations, using other types of tests, if possible, without the presence of an interviewer/supervisor and offering support based on new technologies (Internet videos, for example). Free telephone helplines are also needed to provide support and information on how to confirm the test result and receive follow-up care, if required. These helplines already exist in many countries, including Spain.

Evidence on the feasibility of self-testing is a prerequisite if policy makers involved in HIV testing are to consider this over-the-counter technology as an additional tool that could help promote early diagnosis, although the decision must obviously take into account numerous other factors in terms of benefits, drawbacks, and potential impact.
